# Acupuncture for Poststroke Dysphagia: A Pilot, Nonrandomized, Self-Controlled Trial

**DOI:** 10.1155/2020/4689296

**Published:** 2020-05-11

**Authors:** Yu Tat Chan, Hong Wei Zhang, Wai Zhu Sun, Kevin Ka Hang Or, Yuan-Qi Guo, Min Chen, Guan-Yi Wu, Guang-Yao Xu, Connie Leung, Sylvia Tam, Francis Chun-keung Mok, Yiu Keung Kwan, Eddie Chow, Joshua Kam Wo Mak, Angus Chun-Kwok Chu, Kathy Lee, Thomas Law, Rita Wai Ming Wong, Zhi-Xiu Lin

**Affiliations:** ^1^School of Chinese Medicine, Faculty of Medicine, The Chinese University of Hong Kong, Shatin, Hong Kong; ^2^Torrens University Australia, Melbourne, Victoria, Australia; ^3^Pok Oi Hospital-The Chinese University of Hong Kong Chinese Medicine Centre for Training & Research, Shatin, Hong Kong; ^4^Pok Oi Hospital-The Chinese University of Hong Kong Chinese Medicine Centre for Training & Research, Yuen Long, Hong Kong; ^5^Yan Oi Tong-The Chinese University of Hong Kong Chinese Medicine Centre for Training and Research, Tuen Mun, Hong Kong; ^6^Tuen Mun Hospital, Hospital Authority, Tuen Mun, Hong Kong; ^7^The Institute of Human Communicative Research, Department of Otorhinolaryngology, Head and Neck Surgery, Faculty of Medicine, The Chinese University of Hong Kong, Shatin, Hong Kong

## Abstract

**Objective:**

To evaluate the effectiveness and safety of acupuncture treatment for dysphagia as a complication of stroke. *Methods and Design*. This is a multicenter, pragmatic, nonrandomized, self-controlled clinical trial. A total of 39 patients were recruited from several Chinese medicine outpatient clinics and hospital-affiliated speech therapy outpatient clinics in Hong Kong. 26 patients completed all the 24 sessions of acupuncture treatment within two months, and only 12 of them were used as self-control. For the self-control group, the retrospective clinical data was taken from the electronic patient records with patient consent. The descriptive swallowing function data were converted into the quantitative Royal Brisbane Hospital Outcome Measure for Swallowing (RBHOMS) scores by two registered speech therapists through a validation process. And the data were validated by reaching consensus between the two speech therapists. All subjects underwent a baseline assessment before commencement of treatment, and outcome assessments were conducted upon the completion of treatment. The primary outcome measure is the RBHOMS score, which is a swallowing disability rating scale for monitoring difficulties in daily swallowing function. Secondary outcome measures include the Chinese version of the Swallow Quality-of-Life Questionnaire and adverse events. All the primary and secondary outcomes were assessed at baseline as well as at the end of acupuncture treatment (month 2).

**Results:**

A total of 39 participants aged 46 to 89 years were enrolled in the study, and the male-to-female ratio was 15 : 11. The mean baseline RBHOMS score of all 39 participants was 5.92 ± 2.23. The mean retrospective RBHOMS score of the 12 subjects who were used as self-control was 5.67 ± 1.72 before enrollment, while the mean RBHOMS score of the 26 participants who completed all the 24 sessions of treatment was 6.92 ± 2.07. There were statistically significant differences between the RBHOMS score at the completion of treatment and baseline (*p*=0.006), and retrospective data (*p*=0.042). Moreover, a significant difference was also found in terms of swallow quality-of-life score before and after acupuncture treatment (*p* < 0.01).

**Conclusions:**

This pilot study provides preliminary evidence for the effectiveness of acupuncture for poststroke dysphagia. The findings from this trial can be used as a foundation for future full-scale randomized controlled clinical trials to assess the efficacy and safety of acupuncture for poststroke dysphagia. *Ethics and Dissemination*. The ethical approval of the clinical research study was granted by the Research Ethics Committee of both New Territories East and West Cluster of Hong Kong. Written informed consent was obtained from all participants, and the study was undertaken according to the ICH-GCP Guidelines. *Trial Registration*. This trial is registered with ChiCTR-TRC-12002621 and the registration date is 2012-10-26.

## 1. Introduction

Dysphagia, or swallowing difficulty, is one of the most common complications of stroke patients [1, 2]. Impairment in cerebral, cerebellar, or brain stem after stroke can impair swallowing physiology [3]. The reported incidence for poststroke dysphagia ranges widely from 29% to 81% due to different methods of diagnosis, time after stroke, and types of lesions [3, 4]. Previous studies suggested that dysphagia may spontaneously resolve in a large proportion of patients during the first 7 days after the cerebrovascular event; however, around 10% of them may develop swallowing problems in six months after stroke [5, 6]. Poststroke dysphagia is known to be associated with an increased risk of pneumonia, malnutrition, disability, and mortality [3, 7, 8] and may also increase the chance of recurrent stroke and prolonged hospital stay [9].

Many management options are available for dysphagia today, including texture-modified diets, swallowing therapy programs, nonoral feeding, medications, and physical stimulation. However, only limited clinical evidence has been demonstrated for the roles of routine management in poststroke dysphagia patients based on a series of systematic reviews [10–12].

Acupuncture is widely employed in the management of poststroke complication in the Asia and Pacific region. According to a report published by World Health Organization (WHO) in 2003, stroke is one of indications of acupuncture [13]. A systematic review, which included about 35 randomized controlled trials of acupuncture for dysphagia, has shown that acupuncture may improve swallowing function [14], while another updated systematic review, which included 29 randomized controlled trials of acupuncture treatment for poststroke dysphagia in short term, demonstrated superior efficacy compared with rehabilitation or medication [15]. Some mechanistic studies have also illustrated that acupuncture is able to regulate the cortex and the swallowing center of the reticular structure of the brain stem to control swallowing reflection and coordinate motor movement of the swallow-related muscles, as well as directly improve the recovery of the injured peripheral nerves [16, 17]. Other studies have also found that acupuncture treatment could attenuate the plasma endothelin and nitric oxide (NO) levels, regulate the imbalance between prostacyclin and thromboxane A2, and improve blood viscosity, thereby collectively contributing to the therapeutic effect for poststroke dysphagia [18–20].

Due to some methodological limitations, such as small sample size, inappropriate outcome measures, methodology and reporting flaws, lack of blinding in outcome assessment, and possibility of publication bias, few previous studies could provide sound evidence for supporting the clinical application of acupuncture or not [21–23]. In the past few years, our research group has successfully conducted a pilot clinical study, which has demonstrated some encouraging results to show the promising acupuncture treatment effect for poststroke dysphagia. To further examine the effectiveness and safety of acupuncture for the treatment of poststroke dysphagia, we conducted an assessor-blinded, self-controlled clinical study.

## 2. Methods

### 2.1. Overall Study Design

The study was conducted in three Chinese medicine clinics of the Chinese University of Hong Kong Clinical Centre for Teaching and Research in Chinese Medicine: the Pok Oi Hospital (Shatin and Yuen Long) and the Yan Oi Tong (Tuen Mun). A total of 39 patients who were diagnosed as poststroke dysphagia between January 2014 and April 2016 were included, and the patients were recruited from Chinese medicine clinics and outpatient speech therapy clinics in the Prince Wales Hospital as well as Geriatric Day Hospital in Tuen Mun Hospital. This project was approved by both the Joint Chinese University of Hong Kong New Territories East Cluster Clinical Research Ethics Committee (CRE-2012.236-T) and New Territories West Cluster Clinical Research Ethics Committee (NTWC/CREC/1281/14). Written informed consent was obtained from each participant. The study was conducted in accordance with ICH-GCP Guidelines as well as the Declaration of Helsinki.

**Figure 1:**
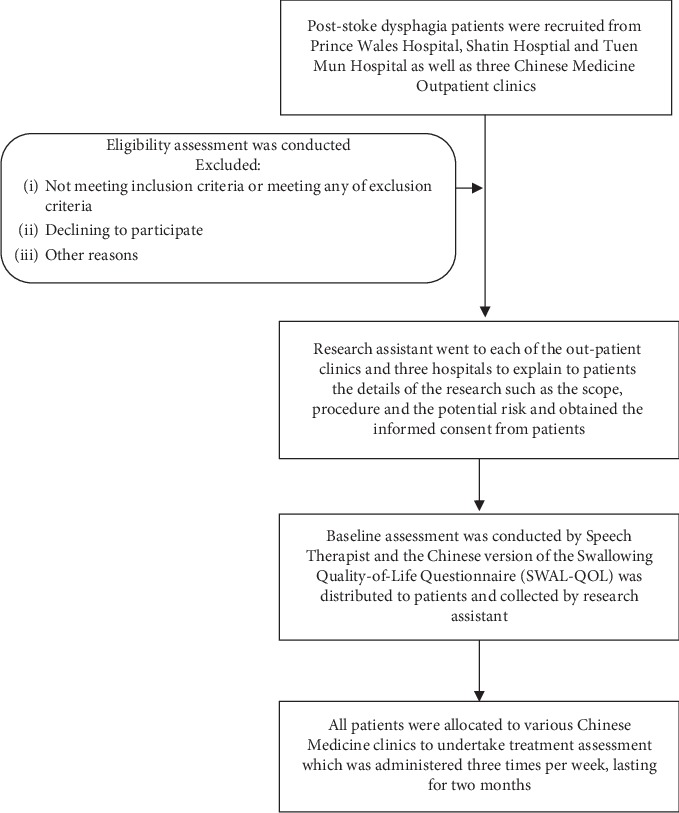
The workflow of the study.

As our research team confronted enormous difficulties in conducting a full-scale randomized control trial of acupuncture for poststroke dysphagia in Hong Kong healthcare setting such as insufficient number of eligible patients, noncompliance of treatment protocol, and high dropout rate, the self-controlled approach was utilized in this pilot study. The study workflow is shown in Figure 1.

### 2.2. Inclusion and Exclusion Criteria

Inclusion criteria were (1) age between 30 and 90 years; (2) having an ischemic or hemorrhagic stroke confirmed by a CT scan; (3) being within 12 months after the first or any recent stroke (ischemic or hemorrhagic); (4) being on modified diet due to dysphagia fulfilling RBHOMS with 1–9 points; and (5) ability to give informed consent (either by patient himself/herself or by caretaker).

Exclusion criteria were (1) dysphagia caused by head injury or neurological disease other than stroke; (2) unstable cardiac arrhythmia; (3) life threatening infection; (4) unconsciousness or severe cognition deficits; (5) oral or throat diseases that display dysphagia; (6) patients with bleeding tendencies or thrombocytopenia, who are taking anticoagulants or have advanced malignancy; and (7) patients with tracheostomy.

Patients who fulfilled all inclusion criteria and none of the exclusion criteria were recruited in this study.

### 2.3. Interventions

All patients received the conventional supportive and rehabilitative treatment, including swallowing therapy program and physical stimulation. On top of the supportive treatment, patients would also receive real acupuncture treatment. The acupuncture treatment was administered once every two days, three times a week, and the whole treatment period lasted for 2 months (8 weeks). A total of 24 acupuncture treatment sessions were carried out on each patient by three acupuncturists who were registered Chinese medicine practitioners in Hong Kong, having at least 3 years of clinical experience in acupuncture practice.

#### 2.3.1. Underlying TCM Principle and Acupoints Selection

Based on TCM theory, the pathogenesis of poststroke dysphagia is due to the insufficiency of the liver and kidney, the stagnant qi and blood that fail to nourish the tongue, and pathogenic factors, notably wind and phlegm, that further obstruct the meridians and the throat, resulting in swallowing difficulty. The participants were further categorized into two subtype patterns by a registered Chinese medicine practitioner who was involved in the study, i.e., (1) pathogenic wind phlegm syndrome and (2) liver yang rebellion syndrome. Those patients with dizziness and thin tongue coating belong to the liver yang rebellion syndrome, while those with greasy tongue coating belong to the pathogenic wind phlegm syndrome. All patients received acupuncture treatment at acupuncture points including GB12 (Wan Gu, bilateral), GB20 (Feng Chi, bilateral), CV23 (Lian Quan), DU20 (Bai Hui), LI4 (He Gu, bilateral), HT5 (Tong Li, bilateral), Hang Sang, Shang Lian Quan (EX-HN20), and Lower 2/5 of the motor zone of scalp acupuncture.

Besides the above regular acupuncture points, additional acupuncture points were used for patients' clinical syndrome subtypes. ST40 (Feng Long, bilateral) was used for pathogenic wind phlegm subtype, and LR3 (Tai Chong, bilateral) for liver yang rebellion type. Standard acupuncture treatment protocol was based on the consensus of our acupuncture expert panel and previous studies [22,23].

#### 2.3.2. Manipulation

Sterile disposable stainless steel needles of various lengths and diameters were used in the study. The standardized needling manipulation methods on different acupuncture points are described as follows: GB12 and GB20 (a depth of 0.8–1.2 cun with the needle tip pointing towards the throat was punctured, and needles were rotated with reinforcing techniques); CV23 (the needle was inserted obliquely to about 1.0 cun towards the root of the tongue, with mild lifting and thrusting technique applied for about 1 min); DU20 (the needle was inserted obliquely at an angle of 30 degrees to a depth of 0.5 to 0.8 cun and rotated for about 30 seconds before being withdrawn); LI4 (the needle punctured perpendicularly for 0.5 to 1.0 cun, followed by applying reinforcing technique for 30 seconds with lifting and thrusting movement); HT5 (the needle punctured perpendicularly to a depth of 0.5 cun, with reinforcing technique, with lifting and thrusting movement applied for 30 seconds); Hang Sang (a needle of 75 mm in length was applied to prick the posterior wall of the throat at the depth of about 0.15 cun 4 times without retaining the needle); Shang Lian Quan (EX-HN20) (the needle was inserted obliquely to a depth of 1.0–1.5 cun towards the root of the tongue; then mild lifting and thrusting technique was applied for 30 seconds); Lower 2/5 of the motor zone of scalp acupuncture (the needle was inserted transversely at 15-degree angle to the skin surface and pushed to a depth of about 1.0 cun, followed by applying twirling method for about 30 seconds); ST40 (the needle was punctured perpendicularly for 1.5 cun, and a reducing technique was applied by lifting and thrusting the needle); LR3 (the needle was inserted perpendicularly for 0.5–1.0 cun, and a reducing technique was applied by lifting and thrusting the needle for 30 seconds).

### 2.4. Outcome Measurements

#### 2.4.1. Primary Outcome Measurement

The Royal Brisbane Hospital Outcome Measure for Swallowing (RBHOMS) [24] was used as the primary outcome measure. RBHOMS is a validated bedside assessment tool with 10 ordinal points over four stages of oral intake. The four stages are stage A, nil by mouth (levels 1–3); stage B, commencing oral intake (level 4); stage C, establishing oral intake (levels 5–7), and stage D, maintaining oral intake (levels 8–10), respectively. All RBHOMS assessments were undertaken by a registered speech therapist. RBHOMS is widely employed to assess the swallowing function of dysphagia patients in daily routine practice.

The RBHOMS data was collected at prebaseline, baseline (pretreatment), and posttreatment interval. Prebaseline RBHOMS scores were extracted from the participants' medical records, according to their latest dysphagia diagnosis and recommendations documented prior to enrolling into this study. These were conducted independently by a speech-language pathologist with more than 10 years of experience working with dysphagia. Pretreatment and posttreatment RBHOMS scores were given immediately after the clinical bedside swallowing assessment conducted at the visit.

#### 2.4.2. Secondary Outcome Measurement

The Chinese version of the Swallow Quality-of-Life Questionnaire (CSWAL-QOL) has 10 scales with 30 items designed to assess eight concepts of dysphagia related to quality of life, including burden, eating duration, eating desire, food selection, communication, fear, mental health, and social functioning and two concepts of generic quality of life, namely, fatigue and sleep. The CSWAL-QOL was selected as a secondary outcome because it is as sensitive, valid, and reliable as the original version [25].

All possible adverse events of acupuncture, including dizziness, bleeding, hematoma, and injury to important organs by acupuncture, were recorded on the Adverse Event Page of the Case Report Form.

In this study, the primary outcome was measured at three time points, i.e., three months before enrollment of the study, baseline assessment, and after the completion of acupuncture treatment, respectively. The secondary outcome measures were collected at baseline and following the end of treatment.

### 2.5. Statistical Analysis

The statistical software of SPSS version 20 (SPSS Inc., Chicago, USA) was used for data analysis. Descriptive statistics were calculated for all the baseline variables and outcome measures. Repeated measures ANOVA was used to analyze change in RBHOMS while paired *t*-test was used to analyze change in CSWAL-QOL score. All the data from dropout subjects were excluded from analysis. All statistical tests were two-sided, and *p* < 0.05 was considered statistically significant.

## 3. Results

**Figure 2:**
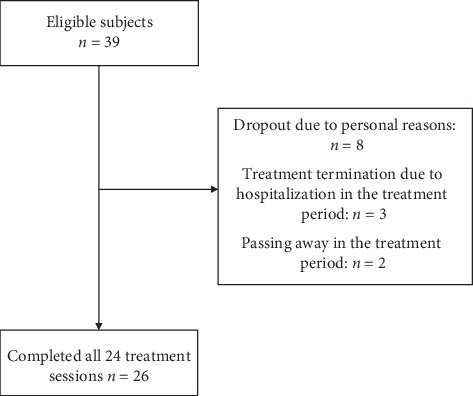
The flowchart of the study according to the CONSORT diagram.

From January 2014 to April 2016, a total of 39 subjects were recruited from various locations.  Figure 2 demonstrates the study flowchart. The mean age of the study subjects was 70.46 ± 11.8 years, ranging from 46 to 89.  Table 1 illustrates the baseline characteristics of all subjects. The male-to-female ratio is 15 : 11. 26 subjects completed the 24 treatment sessions, and 13 patients dropped out due to various reasons, such as inability to attend treatment sessions due to difficulty in arranging transportation, noncompliance with the treatment protocol, and admission to hospital due to stroke complications or other diseases.

All retrospective data of swallowing function from eligible patients was taken from the electronic patient records stored in their respective hospitals. All clinical description of swallowing function in patient record was converted to the RBHOMS score by a registered speech therapist hired by the project. The dataset of RBHOMS score of the patients three months after the onset time of the latest stroke was analyzed. All the retrospective RBHOMS scores in the self-control group were validated by reaching consensus between two speech therapists. The RBHOMS score closest to the recent stroke was taken as self-control data for further analysis.

Only 12 subjects' retrospective data was suitable for using as self-control. The reasons for excluding the retrospective data of the other subjects were missing of clinical data and onset time of the current stroke being beyond three months.

As shown in Table 2, the mean RBHOMS score of these 12 subjects before enrollment in the study was 5.67 ± 1.72. The baseline RBHOMS score of all the 39 patients was 5.92 ± 2.23. At the completion of the treatment, the RBHOMS score improved to 6.92 ± 2.065.  Table 3 shows that there was statistically significant difference between baseline RBHOMS score and that at the completion of treatment (*p*=0.006). Similarly, a marked difference was found between the retrospective RBHOMS score and that at the completion of treatment (*p*=0.042). However, no statistical difference between retrospective and baseline RBHOMS scores was found (*p*=1.000).

With regard to the scale in the CSWAL-QOL, pre- and postanalysis were used. 26 of the subjects were asked to complete the CSWAL-QOL. Paired *t*-test was used for analysis. Significant difference was found in terms of swallow quality-of-life scores before and after acupuncture treatment (*p* < 0.01).

All subjects were asked to complete an adverse report form, on which the potential adverse events, including faintness, dizziness, hematoma at the acupuncture sites, local infection, and injury to important organs, were described. No adverse events were reported in the whole study.

## 4. Discussion

We recruited 39 participants aged 46 to 89 years, of which only 26 completed all the 24 sessions of acupuncture treatment. There were statistically significant differences in the RBHOMS and swallow quality-of-life scores between baseline and end of treatment. For 12 participants with available retrospective RBHOMS data, statistically significant difference between retrospective score and end of treatment was also found; however, no statistical difference between retrospective score and baseline one was found. This indicated that the combination of routine treatment and acupuncture could improve swallowing function as well as symptoms of patients. Moreover, no adverse events were reported in this study, indicating that acupuncture may be a safe treatment option for poststroke dysphagia patients.

Poststroke dysphagia is an important complication of stroke. Acupuncture is widely adopted to treat poststroke dysphagia in China and some other East Asian countries. However, clinical evidence concerning its efficacy and safety is sparse, and it is necessary to evaluate the effectiveness and safety of acupuncture treatment for this common stroke complication to establish clinical evidence. In Hong Kong healthcare setting, it remains very challenging to recruit sufficient poststroke dysphagia patients from the community as new poststroke dysphagia patients normally stay in rehabilitation hospitals where Chinese medicine treatment is usually not allowed. For this reason, in our study, the treatment was carried out in three Chinese Medicine outpatient clinics. Because of the small number of suitable patients recruited, we adopted a pragmatic design for this study.

In this study, all recruited subjects received acupuncture treatment. In our experience, no patient was willing to be in the control group receiving no treatment or placebo, as this patient population is usually at an advanced age with certain degree of physical handicaps, and they need to make great effort to attend the research clinic. Therefore, it is impractical to recruit sufficient patients for the control arm. For this reason, we designed our study as a nonrandomized, retrospective self-controlled trial.

It has been demonstrated that around 50% of poststroke dysphagia patients may recover spontaneously during the first week of stroke [26]. As there is possibility of spontaneous recovery, it is necessary to include a control arm of no treatment to exclude the influence of natural history. However, due to the difficulty in recruiting patients, it was more feasible to conduct a self-controlled study. Although the self-control in this study cannot exclude the possible existence of natural history of poststroke dysphagia after commencing acupuncture treatment, the comparison between retrospective and baseline data demonstrated little difference, suggesting a relatively stable condition. Also the inclusion of self-control is a practical way under certain circumstances such as insufficient subject recruitment. In fact, a self-controlled approach is more appropriate in early points of development of a new clinical treatment [22]. In our study, all electronic clinical records of the subjects were screened by a research assistant and speech therapists. Out of the 26 patients who completed the 24 treatment sessions, only 12 were appropriate for use in self-controlled group analysis with available relevant retrospective data. In self-control group, the retrospective clinical data was recorded and converted into the RBHOMS score by a registered speech therapist. All the retrospective data in self-control group were validated by reaching consensus between the two speech therapists. A Brazilian study reported that the mean of spontaneous recovery of poststroke dysphagia patients is 2.4 months (SD = 2.7) [23]. To eliminate the bias of spontaneous recovery effect, the RBHOMS score three months before enrolling in the study and three months after the onset time of current stroke were analyzed. Regarding primary outcome measures, repeated measures ANOVA was used for comparing the different RBHOMS scores before enrollment, at baseline, and after treatment. As CSWAL-QOL is a subjective, patient-reported outcome, only pre- and postanalysis were conducted.

The selection of acupoints in the study was based on (1) traditional acupuncture theory that usually combines local and distant points, taking into account the patient's syndrome manifestation; (2) reports of relevant clinical studies; and (3) clinical experience of acupuncture experts in our research group. Together, treatment on these acupoints has the effect of tonifying liver and kidney, expelling pathogenic wind and phlegm [21]. The upper-middle line of occiput is also selected for stimulating medulla oblongata. We used the same acupoints for every treatment. In our study, we categorized the patients further into two subtypes of syndromes: those with the syndrome of pathogenic wind and those with pathogenic phlegm. With this subdivision, extra acupuncture points were included in the prescription for different subtypes. ST40 (Feng Long, bilateral) was used in the patients with syndrome of pathogenic phlegm, while LR3 (Tai Chong, bilateral) for pathogenic wind syndrome.

The current study illustrated that acupuncture treatment was able to improve the swallowing function of the poststroke dysphagia patients. However, several limitations existed in the study. Firstly, it was not a proper randomized controlled trial. A randomized control trial is a gold standard to establish clinical evidence for the effectiveness of a therapy. In randomized controlled trial setting, all confounding factors which may hinder the conclusive finding can be minimized by randomization. However, due to practical difficulties such as insufficient patient numbers, a randomized controlled trial was not possible in the current setting, and a nonrandomized study was adopted. Secondly, there is a lack of control group. Owing to the difficulty of patient recruitment and relatively small sample size, retrospective self-control design was used. Thirdly, most of the subjects of this project were stroke sufferers and comorbidity was very common among these patients, which greatly affected the subjects' compliance. In our experience, incentive free Chinese medicine consultation was offered to enhance their compliance throughout the whole study. Fourthly, due to financial constraints, no follow-up assessment was conducted in our study. Lastly, due to budget and manpower constraints, only subjective assessment tools, i.e., RBHOMS and CSWAL-QOL, were used as outcome measures for this study, and more objective assessment tools such as Fiberoptic Endoscopic Evaluation of Swallowing (FEES) was not used.

## 5. Conclusion

The results from this clinical study illustrated that acupuncture treatment was able to improve both swallowing function and quality life of the poststroke dysphagia patients. This pilot clinical study can be a foundation for future full-scale randomized controlled clinical trial to assess the efficacy and safety of acupuncture treatment for stroke patients with dysphagia.

## Figures and Tables

**Table 1:** Demographic and disease characteristics of all subjects.

Characteristics	Treatment group
Age (year, mean ± SD)	70.462 ± 11.8
Gender (male/female)	15/11
Stroke type (ischemic/hemorrhagic)	24/2

**Table 2:** The primary outcome measure (RBHOMS) at different time points of the entire study.

Outcome measure	Self-control group	Intervention group	*p* value
RBHOMS score mean (SD)			
First assessment	5.67 (1.723)	5.92 (2.234)	1.000
Second assessment	5.92 (2.234)	6.92 (2.065)	0.006

**Table 3:** Comparison of RBHOMS and swallowing quality-of-life scores between retrospective data, baseline, and posttreatment.

Parameters	Retrospective (three months before enrollment)	Baseline (month 0)	After treatment (2 months after enrollment)	*p*1	*p*2	*p*3
RBHOMS	5.67 ± 1.723 (3–8)	5.92 ± 2.234 (1–9)	6.92 ± 2.065 (2–9)	1.000	0.006	0.042
Swallow quality-of-life score		131.0 ± 39.9 (44–200)	157.5 ± 31.0 (75–208)		0.000	

*p*1 is the *p* value of pairwise comparison between three months before enrollment and month 0; *p*2 is *p* value of pairwise comparison between month 0 and 2 months after enrollment (completion of treatment); *p*3 is *p* value of pairwise comparison between three months before enrollment and 2 months after enrollment (completion of treatment).

## Data Availability

The data used to support the findings of this study are available from the corresponding author upon request.

## Abbreviations

BMI: Body mass index
RBHOMS: Royal Brisbane Hospital Outcome Measure for Swallowing
TCM: Traditional Chinese medicine
CSWAL-QOL: The Chinese version of the Swallow Quality-of-Life Questionnaire.
